# A Screen-Printed Carbon Nanotube/Epoxy Array for Impact Monitoring in Construction Safety Helmets

**DOI:** 10.3390/s26206557

**Published:** 2026-10-16

**Authors:** Ga-Hyeon Eom, Yu-Jin Jung, Puneet Sagar, Sung-Hwan Jang

**Affiliations:** 1 Department of Smart City Engineering, Hanyang University ERICA, Ansan 15588, Republic of Korea; eomgh0316@hanyang.ac.kr (G.-H.E.); yujin0421@hanyang.ac.kr (Y.-J.J.); psagar@hanyang.ac.kr (P.S.); 2 Department of Civil and Environmental Engineering, Hanyang University ERICA, Ansan 15588, Republic of Korea

**Keywords:** carbon nanotubes/epoxy composite, screen printing, piezoresistive sensor, impact monitoring, construction safety helmet, sensor array, spatial response mapping

## Abstract

Conventional construction safety helmets provide passive protection but do not record impact occurrence or location. Recording this information could support post-impact inspection, motivating the integration of distributed sensing onto the curved helmet shell. This study develops a screen-printed carbon nanotube (CNT)/epoxy sensor array for multichannel impact monitoring. Rheological and electrical characterization guided sensor fabrication, while impact tests examined how CNT loading and sensing-disc geometry affected response magnitude and variability. A 5.0 wt.% CNT formulation with a 10 mm sensing disc was selected and integrated onto the inner shell as a 19-channel array. Channel-specific resistor-substitution calibration enabled resistance-based response analysis. Three independently fabricated arrays detected all 240 crown impacts at nominal energies of 0.2–4.0 J. Across 90 impacts at three off-crown positions, the median angular deviation was 6.2°. These findings demonstrate an approach to extending helmet functionality through low-energy impact detection and regional localization using printed CNT/epoxy sensors.

## 1. Introduction

Struck-by incidents rank second only to falls among causes of death in construction and first among causes of nonfatal injury. Between 2011 and 2021, they accounted for roughly 17% of fatalities and 21.5% of nonfatal injuries [1]. A recent review identifies struck-by incidents, along with falls from height, electrocution, and caught-in or caught-between events, as some of the most severe hazards in the industry [2]. However, struck-by incidents cover a broad range of events, including vehicle- and equipment-related incidents as well as impacts from smaller falling or flying objects. Falling objects alone caused about 27% of struck-by fatalities [3]. Impacts from dropped tools, bolts and construction materials are particularly relevant to helmet-based head protection and monitoring. A single blow to the head can cause concussion or long-term disability. In the United States, nonfatal traumatic brain injury was associated with an estimated USD 40.6 billion in healthcare expenditures in 2016, excluding productivity losses and caregiver costs [4]. Recording the time and approximate shell location of an impact could provide additional information for subsequent assessment. Conventional helmets leave this need unmet: they attenuate impact energy but retain no information on the occurrence, location, or magnitude of the impact itself. Wearable systems developed for construction safety monitoring have primarily focused on physiological and environmental conditions rather than head impacts [5]. A helmet that both attenuates an impact and records its occurrence, approximate location on the shell, and relative magnitude could address this gap [5,6].

Efforts toward such a helmet have proceeded in two directions. The first infers head impact from the kinematic response of the helmet or head, measured by accelerometers and gyroscopes [7,8,9]. Such devices can register the occurrence of an impact and estimate its magnitude, while determining its location generally requires supplementary sensors, inverse models and learning algorithms [7,8]. The second direction situates dedicated sensing elements at the point of contact to transduce force or shock directly and has produced increasingly capable demonstrations. Saikia and Alkhader [10] evaluated a smart-textile impact-sensor matrix on a pendulum rig, Li et al. [11] resolved the stress distribution across a headform with a PVDF piezoelectric array, Kim et al. [12] recovered impact energy on laboratory panels with piezoresistive paint grids at energies as low as 0.07–1.0 J, and Zu et al. [13] conformed a 32-unit triboelectric array to the head by 3D scanning and coupled it to machine-learned injury grading. These studies demonstrate that distributed contact sensing can provide information on both impact magnitude and spatial distribution.

Carrying that capability into a commercial safety helmet encounters constraints that the literature on each transducer class has documented. The stiffness and brittleness of piezoelectric ceramics restrict their application to complex curved surfaces [14,15]. Installation requires poling and secure bonding on the target geometry [12,15], and charge-mode readout typically requires channel-specific conditioning and calibration [11]. These constraints have motivated the use of flexible piezoelectric films and paints, although these approaches can involve lower output and increased sensitivity to environmental conditions [14]. Triboelectric devices remain largely hand-fabricated, a barrier to large-scale application that their developers explicitly acknowledge [13]. Smart-textile constructions face a related obstacle: sensing elements and fabrication routes remain insufficiently standardized [10]. For large-scale helmet deployment, manufacturability becomes a central design constraint.

These constraints may be manageable in laboratory demonstrations but become more important when the technology is integrated into a commercial safety helmet. The sensing layer must be scalable to manufacture and compatible with the existing helmet shell without requiring structural redesign. The characteristics of the target impact events add another challenge. Repeated head impacts have been investigated in sports settings [16,17,18,19,20,21], but those findings do not establish injury thresholds for low-energy impacts on construction helmets. The present study therefore evaluates sensor performance without inferring clinical injury risk. For such events, the response amplitude can provide a relative indication of impact magnitude. The response pattern across the array can also indicate the region of contact. Together, these measurements provide information that can be used to distinguish individual impact events by their relative magnitude and location. The main challenge is not only to detect an impact but also to integrate multiple sensing elements onto the curved surface of a commercial helmet. This integration should avoid the added thickness, geometric mismatch, assembly complexity, and manufacturing burden associated with discrete sensors. A sensing layer is needed that conforms to the helmet shell and can be fabricated at scale. It should also provide a relative indication of impact magnitude and spatial information about the contact region during low-energy impacts encountered in routine site work.

Piezoresistive carbon nanotube (CNT)/polymer nanocomposites offer a route to such a layer. A piezoresistive film converts deformation into a resistance change that can be measured using a low-voltage resistive readout, providing a practical route to multichannel acquisition. The extreme aspect ratio of CNTs, coupled with their intrinsic conductivity, makes them efficient conductive fillers at low loadings [22,23]. Above the percolation threshold, CNTs form a three-dimensional conductive network. Impact-induced deformation can alter the inter-tube spacing and disrupt conductive pathways within this network, resulting in a measurable change in electrical resistance [23,24,25]. CNT content is an important design parameter that affects both sensitivity and stability. The choice of matrix is also important. An epoxy matrix was selected because its relatively high stiffness after curing was expected to couple the deformation of the rigid helmet shell effectively to the CNT network.

Structural composites have long exploited this effect for distributed strain and damage sensing [26,27,28]. Augustin et al. [29] localized impact damage using resistance changes from CNT-modified adhesive films. Subsequent work extended resistance-based damage assessment to low-velocity impacts on glass-fiber laminates with a CNT-coated sensing layer [30]. Screen printing addresses the manufacturing constraint identified above. The process is mature and scalable and can pattern films over large areas without photolithography or vacuum processing [31,32]. It can accommodate the relatively high viscosity of particle-filled pastes that can limit inkjet deposition [33], and it proceeds on a planar flexible substrate that is subsequently brought into conformal contact with a curved surface, so that a printed sensor attaches to an existing shell rather than requiring a redesigned helmet. Wang et al. [34] recently demonstrated screen-printed CNT/epoxy sensors for impact detection and localization on composite structures. Their results suggest that printed piezoresistive arrays could provide a low-complexity approach to spatial impact sensing for helmet monitoring.

Such demonstrations have characterized sensors on flat or simply contoured laboratory specimens. Integration onto a curved product surface has received less attention. Such integration requires reliable print uniformity, electrode placement and interconnects. Printed CNT films can accommodate curvature at the single-element level [31,32]. However, it remains necessary to determine whether multiple elements can be distributed over a curved helmet shell and interrogated synchronously while maintaining sufficient channel-level impact contrast. Geometry is also an important design variable. Because the film responds to the strain field generated over the contact region, the sensing-region diameter is related to the size of the impactor. CNT loading and sensing-region size should be considered together when optimizing the impact response. Their combined influence has not been systematically investigated in the context of helmet-shell integration. Filler content in particular has typically been fixed from prior formulations rather than examined as a design variable [12]. Few studies have connected material characterization, array design, conformal integration, and real-time readout within a single end-to-end demonstration. Repeated loading can alter the electrical baseline of CNT/polymer sensors through changes in the conductive network [35]. Long-term impact monitoring therefore requires separate assessment of baseline drift and response stability. Integration into a safety helmet also requires verification that the sensing layer and wiring preserve the performance of the modified assembly.

**Figure 1:**
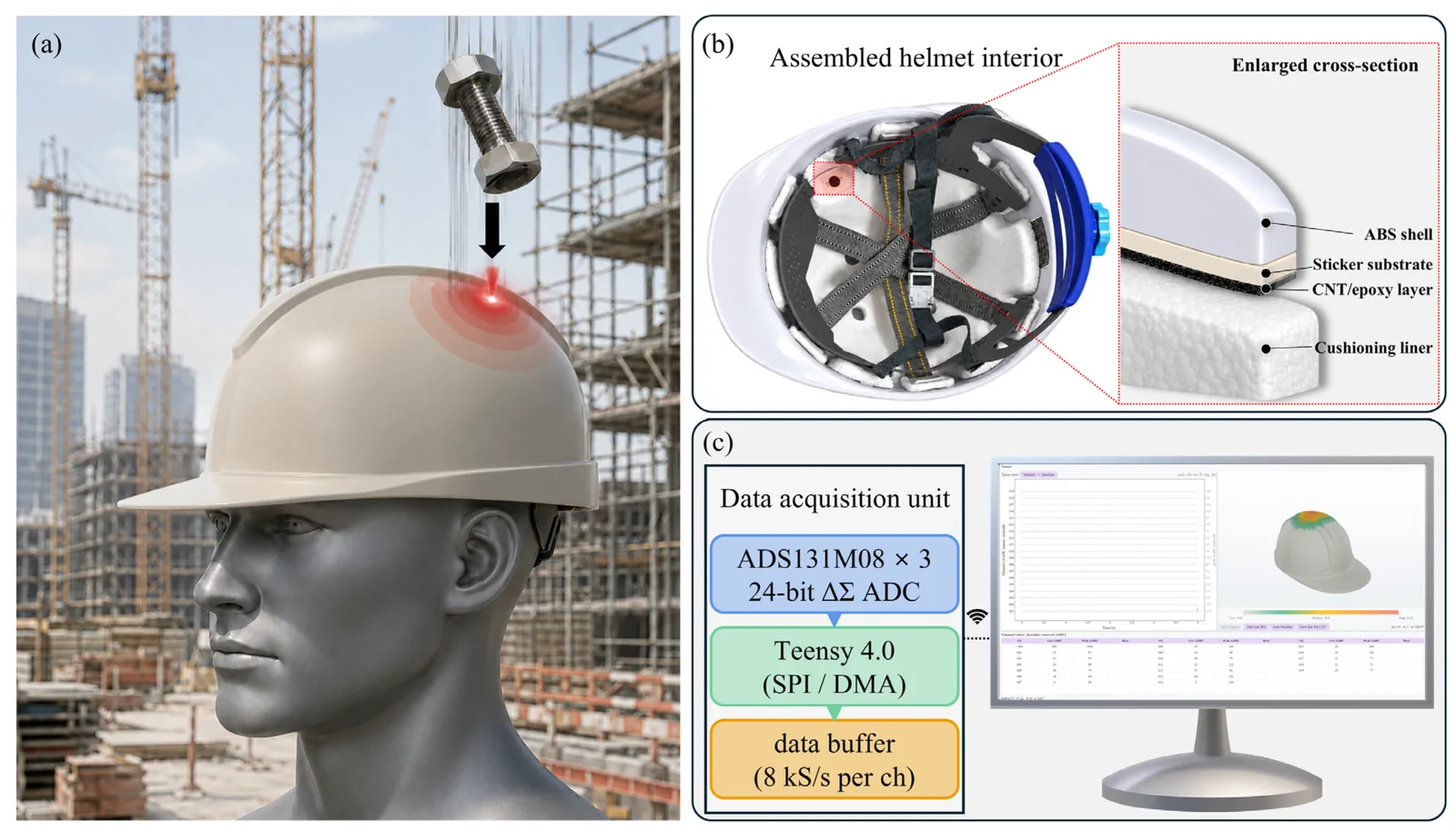
Concept of a screen-printed CNT/epoxy sensor array integrated into a construction safety helmet. (**a**) Illustration of a falling-object impact. (**b**) Helmet interior and an enlarged schematic cross-section of the sensor placement; the black circular features represent the printed CNT/epoxy sensing regions, each with a diameter of 10 mm; the printed layer is approximately 0.2 mm thick. (**c**) Conceptual data acquisition and monitoring interface.

This study addresses that gap with the impact-monitoring system shown in Figure 1, in which the array is treated as a printed sensing layer rather than as a collection of discrete sensors. A screen-printed CNT/epoxy piezoresistive sensor array converts a localized impact on a commercial construction safety helmet into spatially resolved resistance signals during a falling-object impact (Figure 1a). Each sensor pairs a circular sensing region with integrated wing-shaped current paths, forming a compact, screen-printable layout (Figure 1b) that is fabricated on a planar flexible substrate and then attached to the inner shell. The circular sensing region avoids introducing a preferred in-plane direction into the sensor geometry. The impact response of individual sensors is first characterized across CNT loading and sensing-region size, and an operating design is selected on that basis, one that balances response magnitude, variability and energy-response consistency rather than maximizing raw sensitivity.

The selected sensors were arranged into a 19-channel array and interrogated by a simultaneous-sampling acquisition system (Figure 1c). Single-sensor and additional helmet-array tests covered nominal impact energies of 0.2–4.0 J. These tests evaluate impact detection and relative response magnitude; they do not replace helmet protective-performance testing [36,37]. Three independently fabricated assemblies were examined to assess reproducibility beyond a single prototype. The study links material characterization, sensor design, curved-shell integration, and spatial response mapping. The resulting system is intended to record impact occurrence and approximate location, while its amplitude provides a relative response measure rather than a calibrated estimate of delivered impact energy. Field deployment would additionally require validation of repeated-use stability, spatial coverage, and the protective performance of the modified helmet.

## 2. Materials and Methods

### 2.1. Materials

A commercial two-part epoxy mortar (DH-EM80; Daehwa, Seongnam, Republic of Korea; compressive strength: 95 MPa; bond strength: 9.3 MPa) was used as the polymer matrix, consisting of a gray base resin and a brown hardener. Multi-walled carbon nanotubes (MWCNTs; K-Nano 210T, Kumho Petrochemical, Seoul, Republic of Korea) were used as the conductive filler. The bundled nanotubes were synthesized by chemical vapor deposition (CVD) and had nominal outer diameters of 12.1 nm (7–20 nm), bundle lengths of 40–50 µm, a Brunauer–Emmett–Teller (BET) specific surface area of 250–280 m^2^ g^−1^, a carbon purity of 95 wt.%, and a Raman intensity ratio *I*_G_/*I*_D_ ~ 0.9–1.2 as specified by the manufacturer. Acetone (≥99.0%, CAS 67-64-1; Daejung Chemicals & Metals, Siheung, Republic of Korea) was used as the solvent. All materials were used as received without further purification.

### 2.2. Fabrication of the Screen-Printed CNT/Epoxy Sensors

The CNT/epoxy ink was prepared by a solvent-assisted ultrasonication process adapted from previously reported CNT/polymer composite protocols [34,38,39]. Acetone was used as a volatile diluent to reduce the viscosity of the epoxy base resin and to disperse the MWCNTs prior to solvent removal. For each batch, 15 g of base resin was diluted with approximately 50 mL of acetone in a glass beaker and stirred manually until a homogeneous solution was obtained. A predetermined mass of MWCNTs was then added and gently stirred to wet the powder. Throughout this study, the MWCNT loading was defined as the mass of MWCNTs divided by the combined mass of base resin and hardener (excluding acetone), and loadings from 0 to 7 wt.% were investigated.

**Figure 2:**
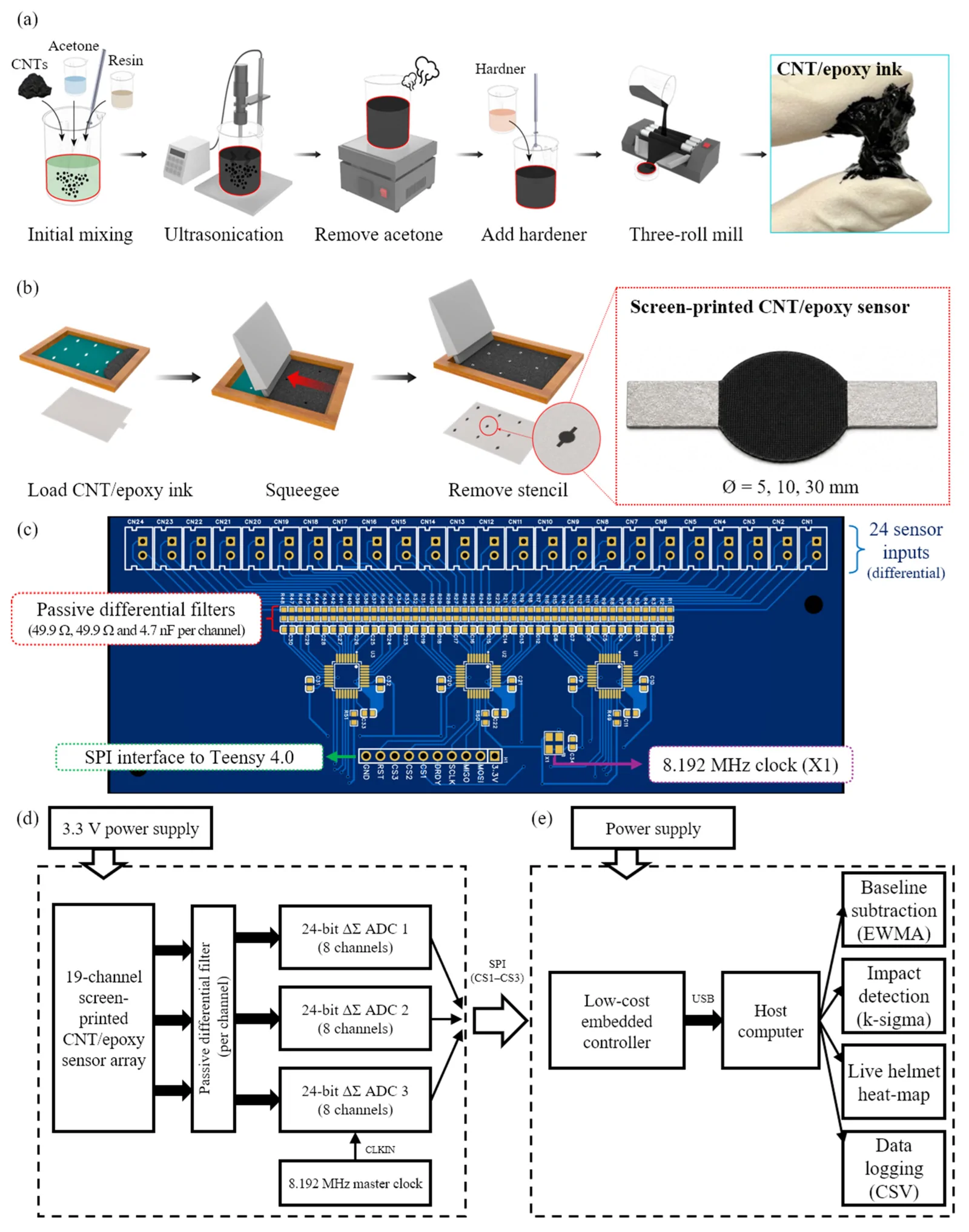
Sensor fabrication and data acquisition hardware. (**a**) Preparation of the CNT/epoxy ink. (**b**) Screen printing of the sensor; the thick red arrow in the squeegee step indicates the direction of squeegee travel. (**c**) Data acquisition board. (**d**) Block diagram of the signal acquisition chain. (**e**) Block diagram of the data processing chain.

The resulting suspension was dispersed with a probe ultrasonicator (Q700, Qsonic, Newtown, CT, USA) fitted with a 12.7 mm diameter tip, operated at 90% amplitude in pulsed mode (15 s on, 15 s off) for a total sonication time of 80 min; the total energy delivered to each batch was approximately 100,000 J. The beaker was immersed in an ice bath during sonication to limit temperature rise and reduce acetone evaporation. This dispersion was then transferred to a hot plate and held at 60 °C for 24 h to remove residual acetone, yielding the CNT/epoxy precursor. Because the mixed two-part system has a working life of approximately 30 min at 23 °C, the hardener was added immediately before screen printing at a base-to-hardener mass ratio of 3:1. The mixture was then passed three times through a three-roll mill (TR50M, Trilos, San Ramon, CA, USA) at 200 rpm to break up residual agglomerates (Figure 2a).

Sensing elements were patterned using a single screen-printing step, based on the established suitability of screen printing for CNT-based conductive films on flexible substrates [31,40]. The CNT/epoxy ink was placed on a patterned 150-mesh polyester screen (db print, Seoul, Republic of Korea). Then, a squeegee was drawn across the screen to force the ink through the open pattern onto the substrate. The screen was subsequently lifted, which leaves the patterned film on the substrate (Figure 2b). The 150-mesh screen accommodates the high viscosity of the CNT/epoxy ink and the coarse MWCNT bundles [40,41,42,43], providing sufficient ink transfer per pass while maintaining pattern definition. Mesh count also sets the deposited thickness and the surface morphology of the printed film, which was characterized separately for a screen-printed CNT/polyurethane system [44]. Each element is a single winged disc: a central circular sensing area flanked by two integral wing-shaped contact tabs. Circular sensing regions of 5, 10 and 30 mm diameter were printed to assess the effect of sensor size. The printed films were cured at room temperature (23 °C) for 24 h. Then, silver paint (RS PRO Silver Conductive Paint, Stock No. 186-3600, RS Components, Corby, UK) was applied to the two wing tabs to form the electrical contacts. The sensors were dried at room temperature for an additional 24 h (Figure 2b).

### 2.3. Fabrication of the Multi-Channel Helmet Monitoring System

The selected sensor design (Section 3.2) was deployed as a 19-channel array on the inner surface of a commercial construction safety helmet (SAHM-1702; SSEDA, Busan, Republic of Korea; ABS shell, radius *R* ≈ 90 mm). The printed elements remained on the adhesive label sheet (LM-3130, Formtec, Paju, Republic of Korea) on which they were patterned, and the printed sheet was attached directly to the inner ABS surface without a separately added adhesive layer. The sensors occupied the existing space between the ABS shell and the internal cushioning layer.

The sensors were arranged in a layout in spherical coordinates (*θ*, *φ*): one crown sensor (*θ* = 0°), an inner ring of six (*θ* = 20°), a mid ring of eight (*θ* = 40°), and four lateral sensors (*θ* = 60°). The printed sensing layer is approximately 0.2 mm thick at the selected condition, and the complete 19-channel array, including the wing-shaped electrodes, adds less than 1 g to the shell, excluding the wiring harness and readout board. The combined area of the nineteen 10 mm circular sensing regions was approximately 14.9 cm^2^, excluding the contact tabs. Sensor centers extended from the crown to *θ* = 60°.

The monitoring system comprises the helmet-mounted sensor array, an analog front end on a custom read-out board, and a host computer that tracks the baseline and displays the result (Figure 2c–e). Each of the nineteen channels passes through a passive differential filter and is then digitized by one of three simultaneously sampling 24-bit ΔΣ converters (ADS131M08, Texas Instruments, Dallas, TX, USA), which provide eight channels each; 19 of the 24 available differential inputs are used and the analog section is operated from a 3.3 V supply (Figure 2c,d). Each sensor is wired to the board through its own two-pin connector and presented across one differential input pair (AINxP/AINxN) of a converter. The read-out is resistive: each element is biased through a fixed 100 kΩ reference resistor from the 3.3 V analog supply, forming a voltage divider whose lower leg is the sensor itself. Because the reference resistance is large compared with the 1–10 kΩ quiescent sensor resistance, the divider provides an approximately constant bias current of 30–33 µA over that range. Resistance conversion nevertheless used the full divider relationship rather than a constant-current approximation. All channels operate at a programmable gain of 1, which leaves headroom within the converter’s ±1.2 V differential input range for large impact transients [45]. The filter on each pair is a differential RC network with 49.9 Ω in series with each leg and a 4.7 nF capacitor across the pair, giving a differential-mode corner of approximately 340 kHz. Every converter is locally decoupled with 1 µF and 220 nF at its analog and digital supply pins. The corner lies far above the signal band by design, because band-limiting is performed by the digital decimation filter and the RC network need only reject content near the modulator rate (CLKIN/2 = 4.096 MHz) [45,46]. A common 8.192 MHz crystal oscillator drives the CLKIN input of all three converters through a 22 Ω series resistor at each device, so every channel is sampled synchronously at 8 kSPS; the resulting sub-millisecond resolution is short compared with the multi-millisecond impact pulse and preserves the phase relationship between channels.

The digitized channels are collected over SPI by an embedded controller (Teensy 4.0, PJRC, Sherwood, OR, USA) and streamed to the host computer over USB (Figure 2e). The controller averages eight consecutive conversions per channel to obtain the nominal 1 kSPS logging stream. The host software, custom-written in Python (v3.14.5), supports baseline subtraction using an exponentially weighted moving average (EWMA; *α* = 0.02), impact detection against a six-sigma threshold, heat-map display, and raw-channel logging [47,48,49]. In continuous-update mode, the EWMA incorporates slow signal changes into the baseline estimate, allowing rapid impact responses to be evaluated relative to the evolving baseline. Array peak responses were converted from ADC amplitudes to percentage resistance changes, as described in Section 2.4.

### 2.4. Characterization and Impact Testing

The rheological behavior of the uncured inks was characterized at 23 °C with a rotational rheometer (Discovery HR20, TA Instruments, New Castle, DE, USA; TRIOS software v5.11) using steady-shear flow curves over 0.01–100 s^−1^, oscillatory stress sweeps from 0.5 to 100 Pa at 1 Hz, and three-interval thixotropy tests. The 0.5–100 Pa range was identified as the linear viscoelastic region. The thixotropy test consisted of 120 s at 1 s^−1^, 30 s at 500 s^−1^, and 600 s at 1 s^−1^. Print quality was quantified from optical micrographs by segmenting individual mesh openings in each printed pattern; the micrographs were acquired with a digital microscope (Dino-Lite Edge 3.0, AnMo Electronics, New Taipei City, Taiwan). A total of 306–413 openings were analyzed per substrate. The printed area fraction and its spatial coefficient of variation were then calculated. Surface morphology was examined by field-emission scanning electron microscopy (Quanta 450 FEG, FEI, Hillsboro, OR, USA). Film thickness was measured with a digital caliper (Digimatic, Mitutoyo, Kawasaki, Japan). Electrical resistance (*R*) was measured using a two-point configuration with a source meter (2450, Keithley, Solon, OH, USA). An electrometer (6517B, Keithley, Solon, OH, USA) was used for high-resistance specimens (*R* > 10^9^ Ω). Copper tape and high-purity silver ink were used at the contacts to minimize contact resistance. The d.c. conductivity was obtained as

(1) $$\sigma =\frac{L}{\mathrm{AR}}$$

where *L* is the inter-contact spacing and *A* = *TH* is the conductive cross-section (*T*, film thickness; *H*, strip width). The measured conductivity followed a percolation-type dependence on MWCNT loading, consistent with models for CNT/polymer composites [50,51]. Changes in the conductive network provide a plausible basis for the piezoresistive response, consistent with mechanisms discussed for CNT/polymer sensors [22,23]. Intrinsic conductivity vs. MWCNT loading was measured on uniform rectangular strips, and the dependence on printing passes (1–10) was measured directly on the winged-disc sensors. Each condition used *n* = 5 specimens with three readings per specimen, reported as mean ± s.e.m.

Impact response was characterized using a guided free-fall apparatus. A steel ball (33 mm diameter, 148 g) was released through a 3 m vertical acrylic guide tube from controlled heights of 0.14–2.76 m. Sensors containing 1.5, 3.0, and 5.0 wt.% CNT were tested at nominal impact energies of 0.2, 0.4, 0.6, 0.8, 1.0, 1.5, 2.0, 2.5, 3.0, 3.5 and 4.0 J. The drop height for each target energy was set using *h* = *E*/(*mg*). For each combination of CNT loading, disc diameter, and nominal energy, three separate specimens were tested and each received three consecutive impacts at that energy, giving nine impact events per condition. Each specimen was used at a single nominal energy, so impact history does not carry across energy levels. Sensing-disc diameters of 5, 10 and 30 mm against the 33 mm impactor correspond to sensor-to-impactor ratios *L*_s_/*D*_imp_ of 0.15, 0.30 and 0.91; *L*_s_/*D*_imp_ is used as a relative geometric descriptor only, *D*_imp_ being the impactor diameter and not a measured contact diameter. Impact energies are reported as nominal potential-energy values calculated using *E* = *mgh*. Air resistance and friction within the guide tube may reduce the kinetic energy at impact. Sensor resistance was recorded continuously using a source meter, and the percentage resistance change was calculated using Equation (2),

(2) $$\frac{\Delta R}{R_{0}}(\%)=\frac{R(t)-R_{0}}{R_{0}}\times 100$$

where *R*(*t*) is the resistance at time *t* and *R*_0_ is the pre-impact reference resistance. For the consecutive-impact trace reported in Section 3.2, *R*_0_ was fixed at 1974.256 Ω, measured at *t* = 0.073 s before the first impact, and retained throughout the three-impact sequence. This fixed reference preserves the accumulation of the residual resistance offset. Recording continued for approximately 5.9 s after the third impact.

A structural bolt–nut assembly (0.522 kg) was selected to represent falling construction fasteners and released through a vertical guide tube with the bolt head serving as the impact face. The contact area was the same as in the steel-ball tests. Crown impacts were applied at nominal energies of 0.2, 0.4, 0.6, 0.8, 1, 2, 3 and 4 J. Ten impacts were applied at each energy to each helmet, yielding 240 impacts in total. Nominal energies were calculated using *E* = *mgh* and were applied in ascending order within each assembly. The upper nominal energy of 4 J corresponds to a calculated release height of approximately 0.78 m for this impactor. Equal nominal energies for the steel-ball and bolt–nut impactors do not imply identical contact conditions; response amplitudes are therefore interpreted within each test protocol. Off-crown tests were conducted at three positions in the spherical coordinate system defined in Section 2.3: P1 (*θ* = 20°, *φ* = 30°), P2 (*θ* = 40°, *φ* = 22.5°) and P3 (*θ* = 40°, *φ* = 157.5°). Five impacts were applied at each position and nominal energies of 0.4 and 2 J on each helmet, yielding 90 off-crown impacts. The quantitative detection and localization statistics were derived from this additional three-helmet dataset.

Each readout channel was calibrated by substituting eight reference resistances spanning approximately 1–12 kΩ. The mean of three raw ADC readings at each resistance was fitted to *C* = *G_i_V* + *b_i_*, where *C* is the ADC code, *G_i_* and *b_i_* are the channel-specific gain and offset, and *V* = *V*_s_*R*/(*R*_ref_ + *R*). The divider used *V*_s_ = 3.3 V and *R*_ref_ = 100 kΩ. Four additional resistances per channel, spanning approximately 1.5–9.1 kΩ, were reserved for verification. Voltage was reconstructed from the fitted relation and converted to resistance using *R* = *R*_ref_ *V*/(*V*_s_ − *V*). Relative reconstruction errors were evaluated against the reference resistances.

For the impact-response conversion, *R*_0_ was the mean of three pre-test readings for each helmet and channel, taken with a digital multimeter (DMM6500, Keithley, Solon, OH, USA). The corresponding divider voltage was *V*_0_ = *V*_s_*R*_0_/(*R*_ref_ + *R*_0_). A baseline-corrected peak amplitude Δ*C* was converted to Δ*V* = Δ*C*/*G_i_*, and the peak resistance was calculated from *V*_0_ + Δ*V* using the divider relationship. The percentage response was then calculated using Equation (2). This conversion expresses the peak response relative to the initial measured *R*_0_, with *V*_0_ treated as the operating baseline. This resistance reference is distinct from the continuously updated baseline used for event detection.

Impact detection was evaluated from *z* = Δ*C*/*σ*, where Δ*C* is the baseline-corrected peak ADC amplitude and *σ* is the channel-specific pre-impact noise estimate; an event was detected when a channel exceeded 6*σ*. Angular deviation was calculated as the angle between unit vectors directed toward the recorded estimated position and the prescribed impact position. The estimates summarize the spatial response distribution rather than the physical contact footprint. For the single-sensor comparisons, robust dispersion was expressed as 100 × 1.4826 × MAD/median, and energy-response consistency was summarized by *R*^2^ from a linear fit of condition medians against nominal energy. Dispersion computed after pooling energies includes both energy dependence and variation among repeated impacts.

For the additional three-helmet dataset, each helmet-array assembly was treated as an independent experimental unit. At each nominal energy, the ten crown-impact responses were averaged within each helmet. The three helmet-level means were then summarized by their mean and a two-sided 95% Student’s *t* confidence interval using two degrees of freedom. Off-crown boxplots summarize the individual events at each position and energy, with repeated impacts nested within each helmet. Localization was summarized using median and 95th-percentile angular deviations.

## 3. Results and Discussion

### 3.1. Rheological, Printability, Electrical, and Morphological Characteristics of the CNT/Epoxy Inks

**Figure 3:**
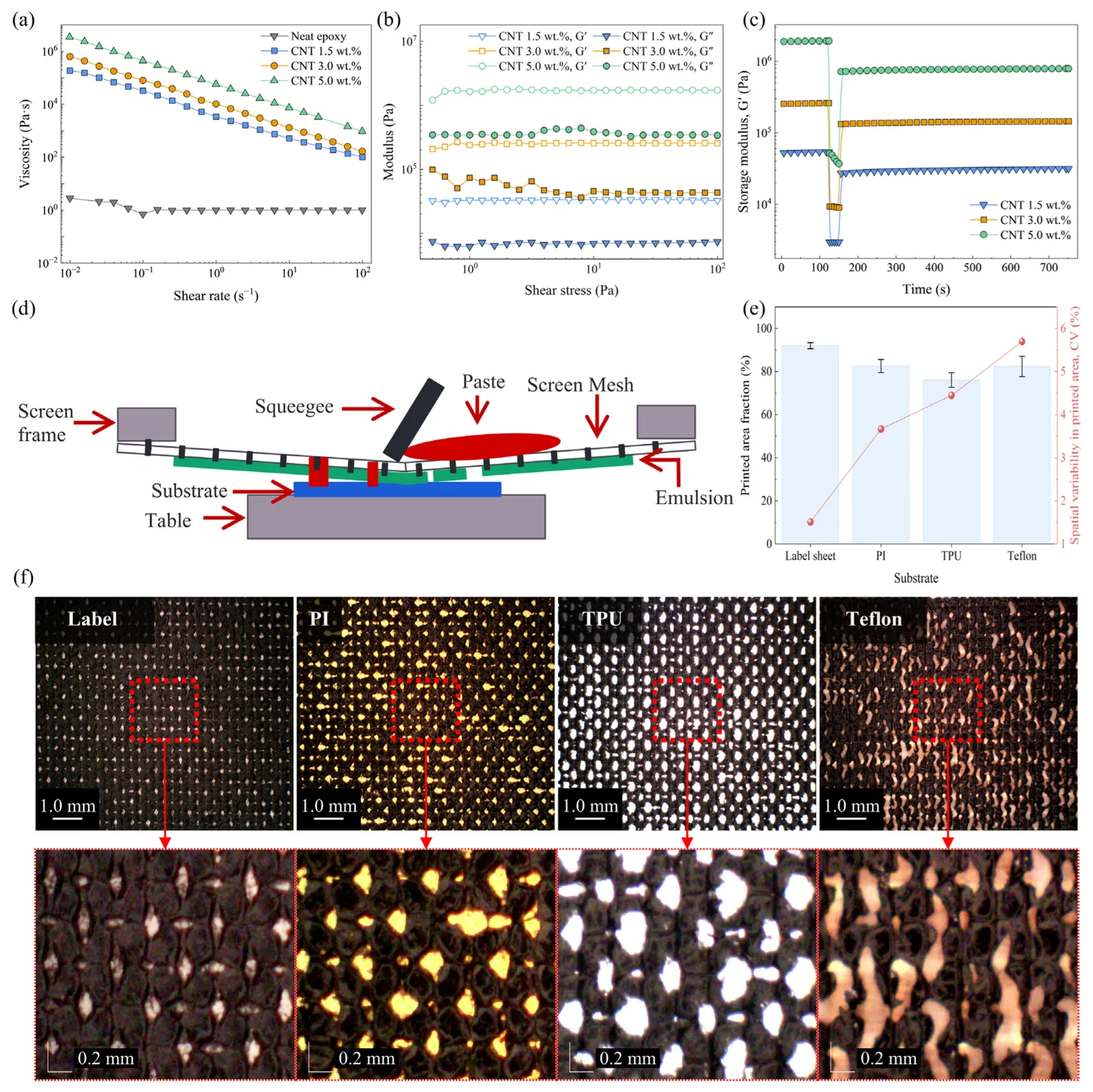
Rheological properties of the CNT/epoxy ink and printing quality on different substrates. (**a**) Viscosity vs. shear rate. (**b**) Storage and loss moduli vs. shear stress. (**c**) Recovery of the storage modulus with time. (**d**) Schematic of the screen-printing process. (**e**) Printed area fraction and its spatial variability (CV) for each substrate. (**f**) Optical micrographs of the printed patterns on each substrate.

**Figure 4:**
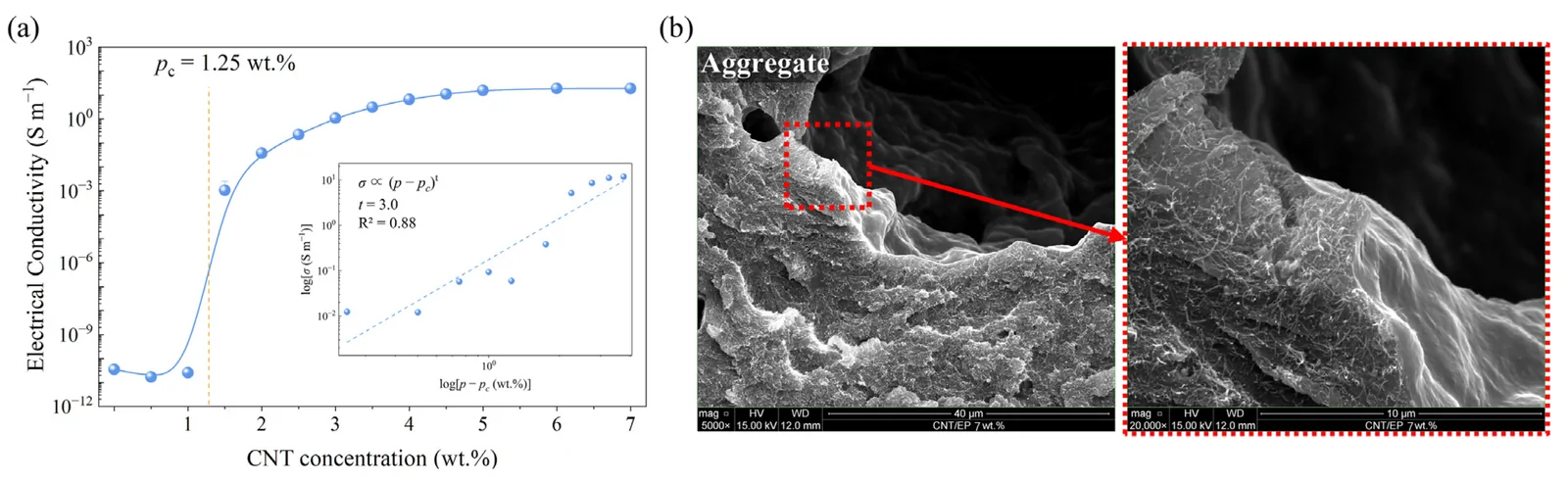
Electrical percolation of the CNT/epoxy composite. (**a**) Electrical conductivity vs. CNT concentration; the vertical dashed line marks the percolation threshold, and the inset shows the power-law fit (dashed line) above the percolation threshold. (**b**) SEM images of the 7 wt.% CNT/epoxy composite at two magnifications; scale bars are 40 and 10 µm.

Figure 3 and Figure 4 relate the processing characteristics of the CNT/epoxy inks to the electrical and morphological properties of the cured composites. In Figure 3a, neat epoxy exhibited nearly Newtonian behavior, with a viscosity of approximately 1–2 Pa·s over most of the measured range. CNT addition produced pronounced shear thinning: the viscosity of the 1.5 wt.% ink decreased from approximately 10^5^ to 10^2^ Pa·s as the shear rate increased from 0.01 to 100 s^−1^. Increasing CNT loading raised the low-shear viscosity, which reached approximately 10^6^ Pa·s at 5.0 wt.%. The concentration dependence is consistent with stronger interactions among CNTs, while their rearrangement and alignment under flow provide a mechanism for the decrease in viscosity [52,53].

The oscillatory measurements show a corresponding increase in elastic response (Figure 3b). The storage modulus, *G*′, increased from approximately 3 × 10^4^ Pa at 1.5 wt.% to 3 × 10^5^ Pa at 3.0 wt.% and 2 × 10^6^ Pa at 5.0 wt.%. Across 0.5–100 Pa, *G*′ and the loss modulus, *G*″, remained approximately constant, with *G*′ exceeding *G*″ by a factor of approximately two to seven. This elasticity-dominated response supports the formation of a mechanically connected CNT structure in the uncured epoxy, consistent with the increase in modulus reported for more strongly interacting CNT suspensions [52].

The three-interval test resolved the response of this structure to a change in shear history (Figure 3c). High shear reduced *G*′ by approximately 18-, 29-, and 52-fold for the 1.5, 3.0, and 5.0 wt.% inks, respectively. After the shear was reduced, *G*′ increased rapidly during the first several seconds and approached approximately 60% of its initial value at 1.5 and 3.0 wt.% and 40% at 5.0 wt.%. The decrease and subsequent recovery of *G*′ are consistent with disruption and rebuilding of the structure supporting the elastic response [53]. Together with Figure 3b, these results show that CNT loading controls both the initial elastic modulus and the extent of structural recovery after shear.

These rheological characteristics are relevant to successive stages of screen printing (Figure 3d). Shear thinning facilitates passage through the mesh under the squeegee, whereas the higher viscosity at low shear and recovery of elastic structure help retain the deposited pattern [54,55]. The combination of flow under shear and structure rebuilding after deposition therefore provides a processing basis for printing the CNT/epoxy sensing elements.

The substrate comparison identifies a suitable surface for applying this process (Figure 3e,f). The label sheet yielded a printed area fraction of approximately 92%, compared with 83% for polyimide (PI), 76% for thermoplastic polyurethane (TPU), and 82% for Teflon. Its spatial coefficient of variation was approximately 1.5%, compared with 5.7% for Teflon. The optical images likewise show more regular, well-defined features on the label sheet. These results connect the substrate choice to the uniformity of the deposited sensing layer, complementing the rheological control of ink transfer [54]. The label sheet was consequently selected for sensor fabrication.

After curing, CNT incorporation produced a pronounced increase in electrical conductivity (Figure 4a). Conductivity rose from approximately 10^−11^ S·m^−1^ at loadings of 1.0 wt.% and below to approximately 10^1^ S·m^−1^ at 5.0 wt.% and above, approaching a plateau of approximately (2–3) × 10^1^ S·m^−1^. The sharp transition between 1.0 and 1.5 wt.% is consistent with the formation of a connected conductive network, as described for CNT/polymer composites through contact and inter-CNT transport [50,56]. The power-law representation in Figure 4a expresses this concentration dependence using the percolation relationship

$$\sigma \propto {\left(p-p_{c}\right)}^{t}$$

where *p* is the CNT loading, *p*_c_ is the percolation threshold, and *t* is the critical exponent. The representation shown in the inset uses *p*_c_ = 1.25 wt.%, *t* = 3.0, and *R*^2^ = 0.88. The power law provides a descriptive representation of the concentration dependence; variation in conductive contacts may contribute to departures from an exact scaling trend [50,56,57]. Tunneling-percolation models relate nonuniversal exponents to the distribution of conductance between neighboring conductive regions [57]. The measured conductivity transition places the 1.5–5.0 wt.% formulations within the electrically active range selected for subsequent impact sensing.

The SEM images of the 7 wt.% composite show CNT-rich regions embedded in the cured epoxy, with entangled bundles visible at higher magnification (Figure 4b). This morphology comprises structures at both the bundle and aggregate scales. Such arrangements are compatible with conductive connections within and between CNT-rich regions, providing a morphological context for the conductivity increase in Figure 4a [50,56].

Taken together, the rheological measurements, substrate comparison, and conductivity results connect material formulation to sensor fabrication. CNT loading controls flow behavior and electrical connectivity, while the label sheet supports uniform pattern transfer. These findings establish the material and processing basis for the impact-response comparisons that follow.

### 3.2. Impact Response and Sensor Design Selection for Helmet Integration

**Figure 5:**
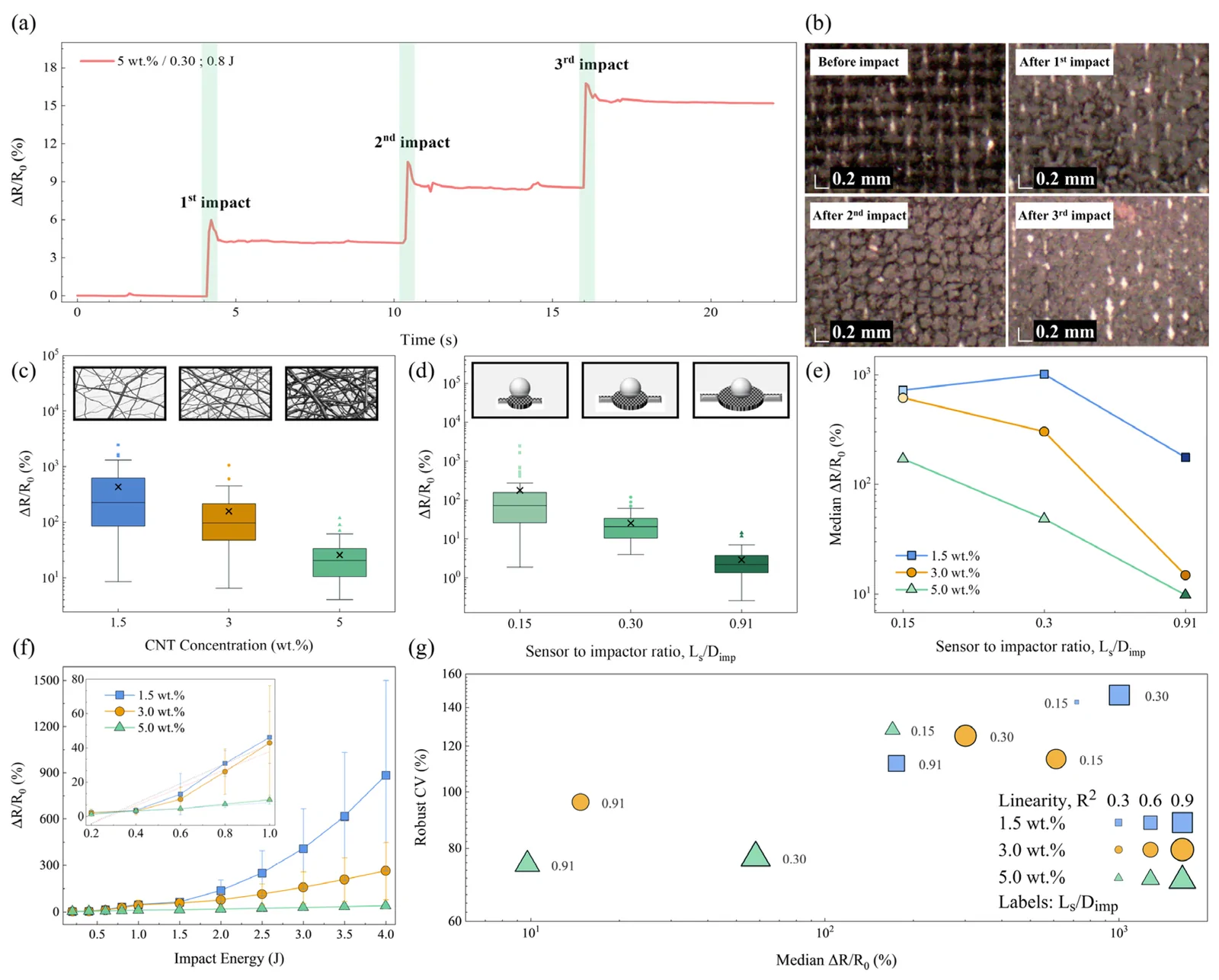
Impact response and design comparison of screen-printed CNT/epoxy sensors. (**a**) Percentage resistance change during three consecutive nominal 0.8 J impacts on a representative 5.0 wt.% sensor with *L*_s_/*D*_imp_ = 0.30, using a fixed pre-first-impact *R*_0_. (**b**) Optical micrographs before impact and after each impact. (**c**) Response distributions for 1.5, 3.0, and 5.0 wt.% CNT at *L*_s_/*D*_imp_ = 0.30, pooled across the plotted energy conditions. (**d**) Response distributions at 5.0 wt.% CNT for three size ratios. In (**c**,**d**), boxes span the interquartile range, internal lines indicate medians, crosses mark means, and whiskers extend to the most extreme observations within 1.5 times the interquartile range of the box edges. (**e**) Concentration–geometry comparison of the plotted median responses. (**f**) Response vs. nominal impact energy, with an inset for 0.2–1.0 J; dotted lines in the inset are linear fits for 1.5 (gray), 3.0 (red), and 5.0 wt.% (blue) CNT. (**g**) Robust CV vs. pooled median response for the nine configurations. Symbol size encodes *R*^2^ from a linear fit of condition medians against nominal energy. Color and symbol shape identify CNT concentration, and labels indicate *L*_s_/*D*_imp_.

Figure 5a shows the response of a representative sensor (5.0 wt.% CNT, *L*_s_/*D*_imp_ = 0.30) to three consecutive impacts at a nominal energy of 0.8 J. Each impact produced a resistance peak followed by partial relaxation, while the residual resistance increased across the sequence. The fixed pre-first-impact *R*_0_ makes this cumulative change visible alongside the individual transients. The positive resistance response is consistent with deformation-induced changes in CNT contacts and interparticle separation, which modify conductive pathways and tunneling resistance [58,59].

The permanent flattening of the mesh-imprinted surface in Figure 5b indicates irreversible deformation of the printed layer. Such deformation can produce persistent changes in CNT contacts and conductive pathways, contributing to the residual resistance after unloading [35]. Further rearrangement of the altered network during subsequent impacts provides a plausible explanation for the progressive residual offset in Figure 5a. For real-time monitoring, impacts are identified from rapid signal changes relative to the local pre-impact baseline (Section 2.3). This approach evaluates each event against the existing signal level without requiring complete recovery to the original baseline.

CNT concentration, sensing-disc geometry, and nominal impact energy were then examined to guide the selection of the array element (Figure 5c–g).

At *L*_s_/*D*_imp_ = 0.30, the median percentage resistance change decreased from 227% at 1.5 wt.% CNT to 97% at 3.0 wt.% and 21% at 5.0 wt.% (Figure 5c). The absolute spread of the pooled responses also narrowed with increasing CNT concentration. These distributions combine energy-dependent changes in response magnitude with variability among impact events, and the concentration-dependent ordering of the medians is apparent despite overlap among individual responses. This trend is consistent with the influence of conductive-network connectivity on piezoresistive sensitivity. At lower CNT loadings, changes in individual pathways can exert a greater influence on the overall resistance, whereas additional contacts at higher loadings can moderate the response to local disruption [58,59].

At a CNT concentration of 5.0 wt.%, increasing *L*_s_/*D*_imp_ from 0.15 to 0.91 reduced the pooled median response from 72% to 2.2%, an approximately 32-fold difference (Figure 5d). The smaller discs also exhibited a broader absolute response distribution. Impact-induced resistance changes depend on the perturbation of current-carrying pathways and the conductive-path arrangement [29,58]. The observed size dependence can therefore be interpreted in terms of the fraction of the sensing network affected by local deformation. A smaller disc may experience perturbation over a larger proportion of its active network, whereas less-affected pathways in a larger disc can moderate the overall resistance change.

The concentration–geometry comparison in Figure 5e shows different response profiles across the CNT formulations. The dependence on disc size is consistent with a response governed by both conductive-network connectivity and the fraction of the network affected by local deformation [29,58]. CNT loading and sensing-disc size thus provide complementary design variables for adjusting the response magnitude of the array element.

The response increased with nominal impact energy from 0.2 to 4.0 J (Figure 5f), with the inset resolving the 0.2–1.0 J interval. At higher energies, the 1.5 wt.% condition showed the largest responses and widest absolute error bars, while the 5.0 wt.% condition retained an increasing response with smaller amplitudes and narrower error bars. This behavior is consistent with greater disruption of the conductive network as impact-induced deformation increases. The stronger response at lower CNT loading may reflect the greater sensitivity of less-connected networks to changes in interparticle contacts and separation [58,59]. The concentration dependence therefore provides a means of adjusting the response range while retaining sensitivity to changes in nominal impact energy.

Figure 5g summarizes the nine configurations in terms of pooled median response, robust dispersion, and energy-response consistency. With three independent specimens per condition, these metrics provide a descriptive basis for selecting among the tested configurations. Considering these characteristics together, the 5.0 wt.% CNT formulation with *L*_s_/*D*_imp_ = 0.30 (a 10 mm sensing disc) was selected for helmet integration. This condition combined a pooled median response of 21% with the moderate response range of the higher-CNT formulation and a compact sensing region. The selected design was subsequently evaluated in additional experiments on three independently fabricated helmet arrays, linking the single-sensor comparison to the array-level results in Section 3.3.

### 3.3. Impact-Magnitude and Spatial Response of the Helmet-Integrated Sensor Array

**Figure 6:**
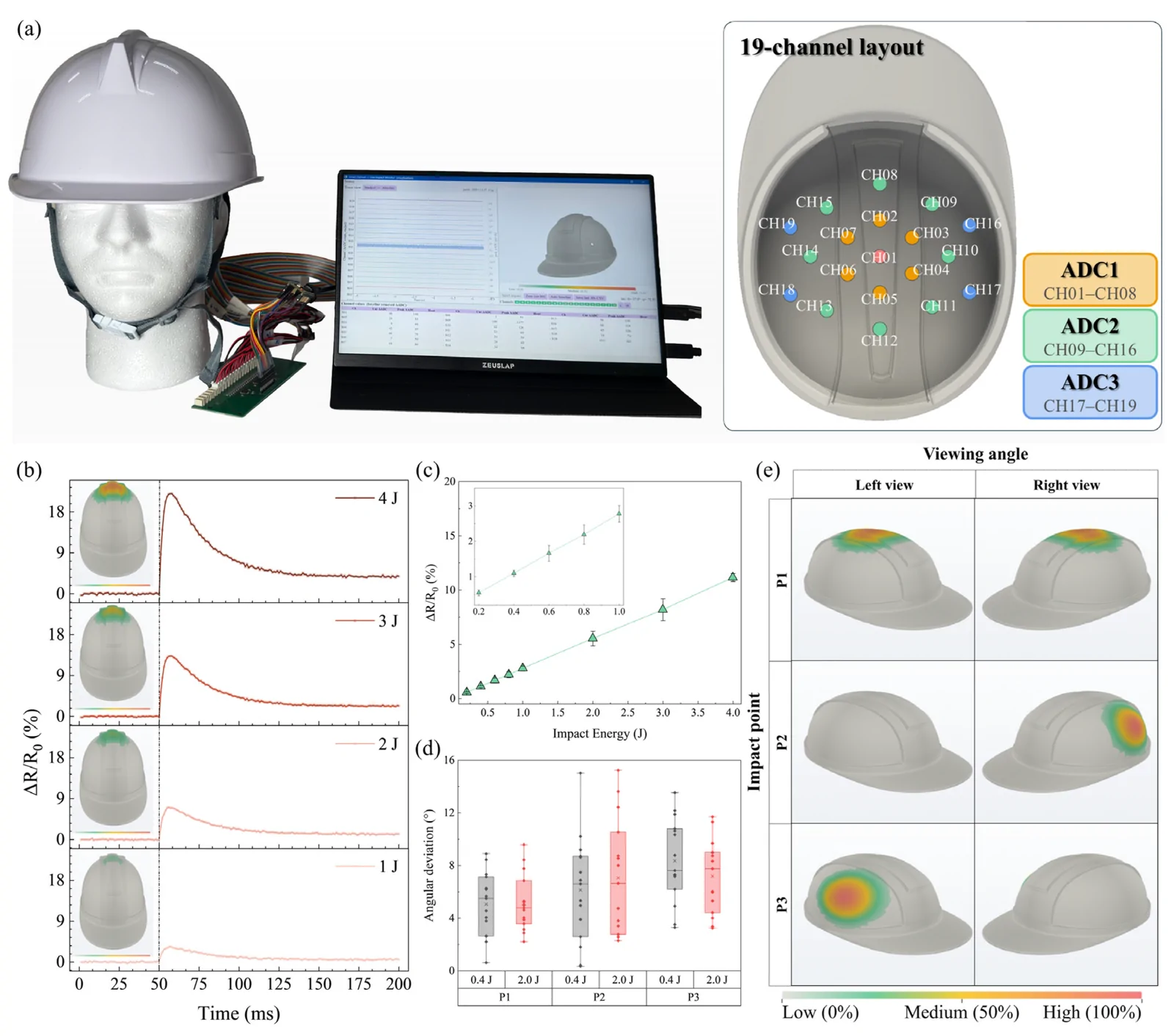
Impact monitoring with the helmet-integrated 19-channel sensor array. (**a**) Representative assembled system and sensor layout. (**b**) Example 1–4 J transient responses with response-map insets (same color scale as in (**e**)). (**c**) Mean peak percentage resistance response vs. nominal energy for three independently fabricated helmet arrays; each helmet-level mean comprises ten crown impacts per energy, and error bars denote two-sided 95% Student’s *t* confidence intervals across the three helmet means (df = 2). The inset shows 0.2–1.0 J. (**d**) Event-level angular deviations at P1–P3 for 0.4 and 2.0 J; each group contains five impacts per helmet on three helmets (15 events). Boxes indicate the interquartile range and the internal line marks the median. (**e**) Illustrative spatial response maps viewed from opposite sides. The heat-map color scale represents normalized display intensity derived from processed channel responses.

The assembled helmet, readout board, and monitoring interface are shown in Figure 6a. Supplementary File S1 presents the drop-test arrangement alongside monitoring-interface replays of crown-impact records at nominal energies of 1 and 4 J. Static resistor-substitution calibration yielded a mean absolute relative resistance reconstruction error of 0.064% and a maximum of 0.457% across 76 verification points excluded from fitting. These verification results establish the accuracy of the channel-specific static resistance conversion used in the readout. All 19 channels remained operational during impact testing, and post-test inspection revealed no visible delamination or edge lifting.

The example waveforms in Figure 6b show a rapid response onset followed by decay. For real-time detection, each impact is identified from the rapid signal excursion relative to the preceding baseline. In continuous EWMA mode, slower changes are incorporated into the baseline estimate [47,48], allowing the transient response to be evaluated against the evolving resistance level. The event-detection signal therefore serves a different purpose from the fixed-baseline analysis in Figure 5a, which retains the residual resistance accumulated during consecutive impacts.

The additional crown-impact tests detected all 240 impacts across the three independently fabricated arrays over the nominal energy range of 0.2–4.0 J. At 0.2 J, all 30 impacts exceeded the 6*σ* detection criterion, with a minimum peak-to-noise ratio of 9.18, demonstrating detection at the lowest energy tested. The mean peak percentage resistance response increased from 0.55% at 0.2 J to 11.15% at 4.0 J (Figure 6c). The corresponding 95% confidence intervals, calculated from the three helmet-level means, were 0.47–0.63% and 10.79–11.51%. These results show an increasing response over the ascending nominal-energy sequence after integration onto the curved helmet shell and confirm impact detection across the independently fabricated assemblies.

Off-crown testing further evaluated spatial localization at three prescribed positions and nominal energies of 0.4 and 2.0 J (Figure 6d). Each position–energy group comprised five impacts on each of three helmets, giving 15 events per group and 90 events in total. The overall median angular deviation was 6.2°, with a 95th percentile of 12.3°, and the group medians ranged from 4.8° to 7.8°. These results extend the array evaluation from crown-impact detection to localization at off-crown positions across multiple independently fabricated assemblies.

Figure 6e presents the spatial display of channel activity from two viewing directions. The colors encode normalized display intensity derived from processed channel responses, with spatial smoothing used to visualize the sampled response field. The paired views facilitate identification of the active region on the curved shell. This display complements the angular-deviation analysis in Figure 6d by presenting the spatial distribution of the measured channel activity. Supplementary File S1 further illustrates the time evolution of the displayed channel responses and heat maps.

Using a spherical approximation with *R* = 90 mm, the median angular deviation of 6.2° and the 95th-percentile deviation of 12.3° correspond to surface arc lengths of approximately 9.7 and 19.3 mm, respectively. This conversion, based on *s* = *Rδ* with *δ* in radians, expresses localization error on the physical scale of the helmet. The measured deviations support identification of the impacted shell region at the tested positions, providing spatial information alongside the event timing and relative response magnitude. Denser sensor placement could provide finer spatial sampling, while its effect on localization accuracy should be evaluated together with the mechanical response field and estimation procedure.

The spatial response can be interpreted in terms of deformation transmitted through the supporting structure. Mechanical deformation can extend beyond a directly loaded element and activate neighboring sensors; array structure and interconnect geometry can consequently influence the spatial response [60,61]. In the present system, the signal from each sensor was acquired through a separate electrical input, and localization used the distribution of channel responses. The off-crown tests therefore evaluate the complete helmet–sensor–readout system, including the mechanical coupling associated with shell integration. Combined with the crown-impact results, this evaluation demonstrates impact detection and regional localization across three independently fabricated arrays.

## 4. Conclusions

A screen-printed CNT/epoxy sensor was integrated onto the inner shell of a construction safety helmet as a 19-channel array. The material and geometry comparisons supported selection of a 5.0 wt.% CNT formulation with a 10 mm sensing region, linking printable ink characteristics to an energy-dependent impact response. Static resistor-substitution calibration yielded a mean absolute relative reconstruction error of 0.064% across 76 verification points. Additional experiments on three independently fabricated arrays detected all 240 crown impacts at nominal energies of 0.2–4.0 J, with the mean peak percentage resistance response increasing from 0.55% to 11.15%. Across 90 impacts at three off-crown positions, the median angular deviation was 6.2° and the 95th percentile was 12.3°. These results demonstrate low-energy impact detection and regional localization using printed sensors integrated onto a curved helmet shell.

Permanent surface deformation was accompanied by an accumulating residual resistance offset, while real-time monitoring evaluated rapid signal changes against the pre-impact baseline. Longer-duration measurements of baseline and sensitivity stability, together with controlled loading sequences, will help distinguish energy-dependent response from impact-history effects during repeated use. Further evaluation across shell locations and fabrication batches will support practical implementation. Quantitative adhesion and environmental durability measurements are needed to extend the attachment observations beyond the reported impact tests. The thin printed sensors were positioned within the existing space between the ABS shell and the internal cushioning layer to limit interference with the original helmet configuration. Preservation of the helmet’s impact attenuation and other protective functions after sensor and wiring integration remains unverified and requires dedicated assessment before deployment.

## Data Availability

The data presented in this study are available on reasonable request from the corresponding author.

## Supplementary Materials

The following supporting information can be downloaded at https://www.mdpi.com/article/10.3390/s26206557/s1, Supplementary File S1 contains a video of the drop-test arrangement and monitoring-interface replays of crown-impact records at nominal energies of 1 and 4 J.
